# Non-Invasive Prediction Models for Esophageal Varices and Red Signs in Patients With Hepatitis B Virus-Related Liver Cirrhosis

**DOI:** 10.3389/fmolb.2022.930762

**Published:** 2022-07-12

**Authors:** Huixin Liang, Hang Si, Mingzhu Liu, Lianxiong Yuan, Ruiying Ma, Genglin Zhang, Jianrong Yang, Zhishuo Mo, Qiyi Zhao

**Affiliations:** ^1^ Department of Infectious Diseases, The Third Affiliated Hospital, Sun Yat-Sen University, Guangzhou, China; ^2^ Guangdong Provincial Key Laboratory of Liver Disease Research, The Third Affiliated Hospital, Sun Yat-Sen University, Guangzhou, China; ^3^ Department of Science and Research, The Third Affiliated Hospital, Sun Yat-Sen University, Guangzhou, China; ^4^ Department of Genetics and Biomedical Informatics, Zhongshan School of Medicine, Sun Yat-Sen University, Guangzhou, China

**Keywords:** liver cirrhosis, hepatitis B, liver diseases, esophageal varices, red signs, prediction model, biomarker, clinical study

## Abstract

**Background:** Red signs are closely related to esophageal variceal bleeding, and, despite improvements in therapy, the mortality rate remains high. We aimed to identify non-invasive predictors of esophageal varices and red signs in patients with hepatitis B virus-related liver cirrhosis.

**Methods:** This retrospective study included 356 patients with hepatitis B virus-related liver cirrhosis after applying inclusion and exclusion criteria among 661 patients. All patients underwent endoscopy, ultrasonography, laboratory examinations, and computed tomography/magnetic resonance imaging. Univariate and multivariate logistic regression analysis were performed, and prediction models for esophageal varices and red signs were constructed.

**Results:** Multivariate analysis revealed that spleen diameter, splenic vein diameter, and lymphocyte ratio were independent risk factors for esophageal varices and red signs. On this basis, we proposed two models: i) a spleen diameter-splenic vein diameter-lymphocyte ratio-esophageal varices prediction model (SSL-EV model); and ii) a spleen diameter-splenic vein diameter-lymphocyte ratio-red sign prediction model (SSL-RS model). The areas under the receiver operating characteristic curve for the two prediction models were 0.843 and 0.783, respectively. With a cutoff value of 1.55, the first prediction model had 81.3% sensitivity and 76.1% specificity for esophageal varices prediction. With a cutoff value of −0.20, the second prediction model had 72.1% sensitivity and 70.7% specificity for the prediction of red signs.

**Conclusions:** We proposed a new statistical model, the spleen diameter-splenic vein diameter-lymphocyte ratio-red sign prediction model (SSL-RS model), to predict the presence of red signs non-invasively. Combined with the spleen diameter-splenic vein diameter-lymphocyte ratio-esophageal varices prediction model (SSL-EV model), these non-invasive prediction models will be helpful in guiding clinical decision-making and preventing the occurrence of esophageal variceal bleeding.

## Introduction

Approximately 240 million people are infected with hepatitis B virus (HBV) worldwide. There are 80 million people with HBV infection in China, of whom 28 million have chronic hepatitis B (CHB). This poses a significant threat to human health. CHB is a major cause of liver cirrhosis (Chen et al., 2018). The incidence of transition from compensated to decompensated cirrhosis is 5–7% per year (D'Amico et al., 2006). As one of the major complications in patients with cirrhosis, esophageal varices (EVs) occur at a rate of 7–8% per year, which may be higher in patients with decompensated cirrhosis (Merli et al., 2003). Esophageal variceal bleeding (EVB) is a serious and life-threatening complication in patients with cirrhosis. The risk of EVB is estimated to be 5–15% per year. The mortality rate of EVB remains around 15–25% at 6 weeks even with active treatment (Garcia-Tsao et al., 2007). Therefore, the effective prevention of EVB is expected to improve the survival rate of patients with cirrhosis.

High risk EVs (HREVs) consist of medium/large EVs and small EVs with red signs in Child C patients (European Association for the Study of the Liver, 2018). As patients with HREVs are more prone to developing EVB, timely prophylactic interventions are recommended. The presence of red signs increases the risk of EVB, regardless of whether the EVs are large or small (World Gastroenterology Organisation, 2014; de Franchis and Baveno, 2015; Garcia-Tsao et al., 2017). Furthermore, the presence of red signs is a risk factor for progression from small to large EVs (World Gastroenterology Organisation, 2014). In patients with Child B/C cirrhosis, small EVs progress to large EVs at a rate of 22% at 1 year and 51% at 3 years (European Association for the Study of the Liver, 2018); thus, the presence of red signs is strongly associated with the occurrence of HREVs and EVB. The prediction of red signs and effective interventions are expected to avoid the occurrence of EVB.

Endoscopy is currently the gold standard for the detection of EVs and red signs (Garcia-Tsao et al., 2017). The Baveno VI consensus recommends regular screening endoscopy in patients with cirrhosis. In compensated patients with no EVs on screening endoscopy, surveillance endoscopy should be repeated every 2 to 3 years (de Franchis and Baveno, 2015). However, endoscopy also has several limitations. It is an invasive examination that may require anesthesia and sedation (Standards of Practice Committee of the American Society for Gastrointestinal Endoscopy et al., 2008), which leads to poor patient tolerance and compliance. There is also the possibility of endoscopy-induced bleeding. Additionally, the relatively high cost of endoscopy limits its popularity in primary medical institutions (Tripathi et al., 2015). Hence, non-invasive, simple, and reproducible methods to predict the presence of red signs are needed in clinical practice, especially in primary medical institutions.

In the last two decades, much effort has been devoted to finding non-invasive methods of predicting the presence of EVs and HREVs. Currently, non-invasive predictive parameters are predominantly imaging/ultrasonographic and laboratory parameters, including spleen diameter, spleen/liver stiffness, portal/splenic vein diameter, platelet count, platelet count to spleen diameter ratio, Fibrosis-4 score (FIB-4), aspartate aminotransferase to platelet ratio index (APRI), and aspartate aminotransferase to alanine aminotransferase ratio (AAR) (Giannini et al., 2003; Giannini et al., 2006; Hong et al., 2009; Kim et al., 2010; Colecchia et al., 2012; Berzigotti et al., 2013; Montasser et al., 2014; Morishita et al., 2014; Rockey et al., 2016). A few studies have reported the non-invasive prediction of EVB (Kothari et al., 2019; Wan et al., 2021) and large EVs (Sharma and Aggarwal, 2007; Lijing et al., 2014). To the best of our knowledge, there is currently no non-invasive prediction method for red signs. Therefore, there is a pressing need to explore non-invasive methods to predict the presence of red signs in addition to EVs.

The objective of this study was to establish non-invasive predictive models of red signs and EVs using imaging, ultrasonography, and laboratory parameters and to evaluate their diagnostic performance. Timely, convenient, and noninvasive prediction of red signs and EVs would benefit clinical diagnosis and therapy, which would help to halt the progression of small EVs and improve the survival rate of patients with HBV-related liver cirrhosis.

## Materials and Methods

### Patients

Among 661 patients with HBV-related liver cirrhosis who were admitted to the Department of Infectious Diseases of the Third Affiliated Hospital, Sun Yat-Sen University between January 2011 and December 2021, a total of 356 patients who met the inclusion and exclusion criteria were finally enrolled in the study. Among the enrolled patients, 224 were patients with decompensated cirrhosis with the presence of decompensating events, and 132 were patients with compensated cirrhosis. A total of 159 (44.7%) patients had a history of antiviral therapy. We obtained an informed consent exemption, approved by the ethics committee of the Third Affiliated Hospital, Sun Yat-Sen University. The study protocol conforms to the ethical guidelines of the 1975 Declaration of Helsinki as reflected in *a priori* approval by the ethics committee of the Third Affiliated Hospital, Sun Yat-Sen University (approval number: [2022]02-62-01).

**FIGURE 1:**
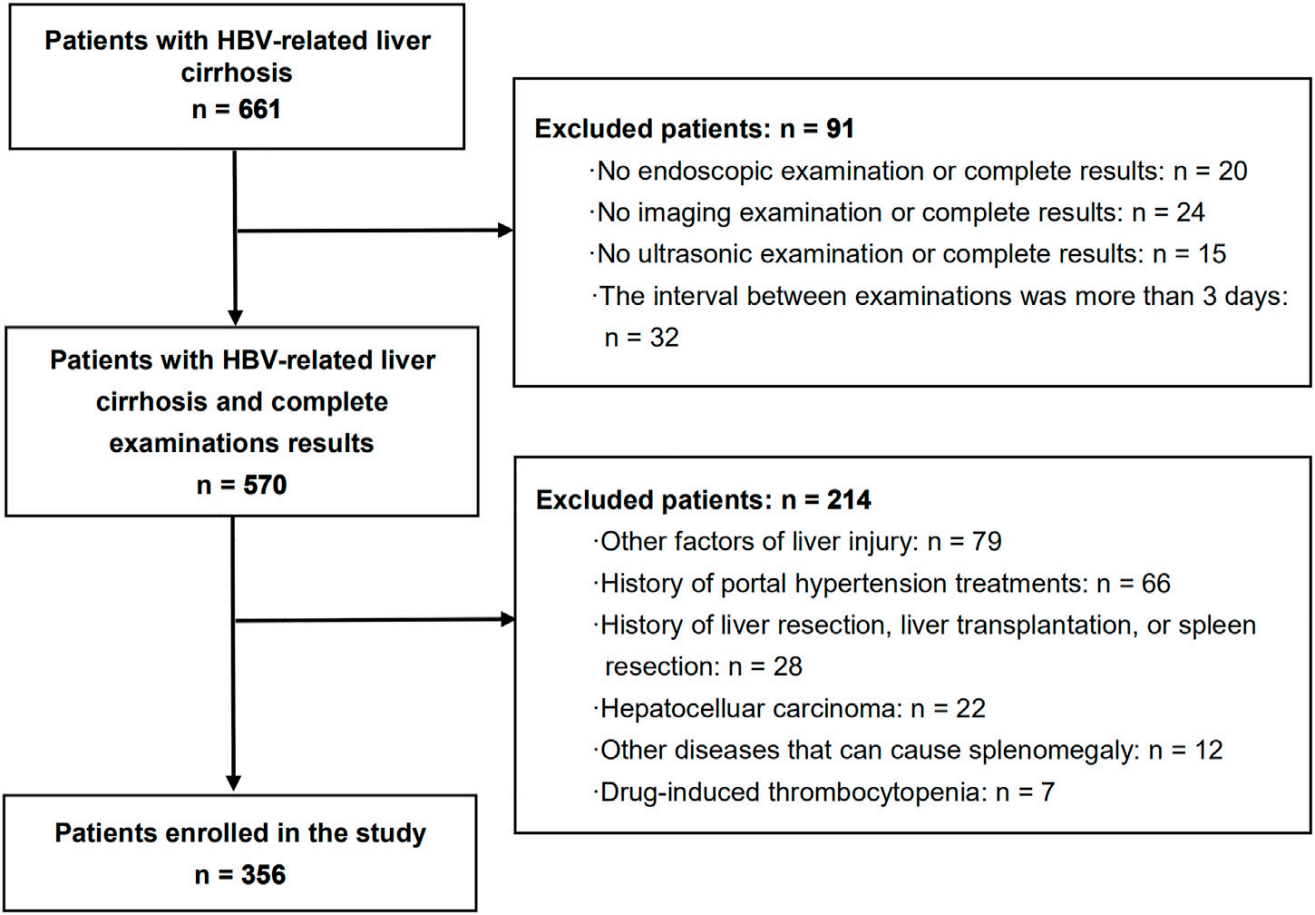
Flowchart of the studied patients.

The inclusion criteria were as follows: (1) positivity for hepatitis B surface antigen for at least 6 months and diagnosis of liver cirrhosis; (2) those that underwent endoscopy, ultrasound, laboratory examination, and computed tomography (CT)/magnetic resonance imaging (MRI), with complete results of all examinations; and (3) the interval between these examinations was not more than 3 days. The exclusion criteria were as follows: (1) other factors of liver injury, such as alcohol, autoimmunity, hepatitis C virus infection, and metabolic liver diseases; (2) history of portal hypertension treatments, such as endoscopic treatments and non-selective beta blockers therapy; (3) history of liver resection, liver transplantation, or spleen resection; (4) hepatocelluar carcinoma; (5) other diseases that can cause splenomegaly, such as leukemia, thrombocytopenic purpura, hemolytic anemia, and multiple myeloma; and (6) drug-induced thrombocytopenia (Figure 1).

### Upper Endoscopic Examination

A standard endoscopic examination was performed by an experienced clinician. Esophageal varices and red signs were diagnosed according to the “Guidelines for the diagnosis and treatment of esophageal and gastric variceal bleeding in cirrhotic portal hypertension” established by the Hepatology Branch of the Chinese Medical Association in 2016.

### Ultrasonic and Imaging Examination

All the patients underwent an ultrasonic examination (MyLab C1 assC Advanced; Esaote, Italy). The splenic vein diameter at the splenic hilum and the portal vein diameter were measured. The presence of ascites was diagnosed. All the patients underwent CT (320 slices Aquilion One Aquilion ONE; Toshiba, Japan) and/or MRI scans on a 3.0T GE system (GE Healthcare, United Kingdom). The spleen diameter was assessed based on the longest splenic dimension measured on CT and/or MRI at the cross-section. Using a double-blind method, two experienced ultrasound physicians and two experienced radiologists independently analyzed the ultrasound, CT, and MRI images. All data from the two independent operators were averaged.

### Laboratory Examination

Laboratory parameters included white blood cell count (WBC), red blood cell count (RBC), platelet count, lymphocyte count, lymphocyte ratio, hemoglobin, prothrombin time (PT), international normalized ratio (INR), creatinine, total bilirubin, albumin, globulin, albumin to globulin ratio (A/G), aspartate aminotransferase (AST), alanine aminotransferase (ALT), alkaline phosphatase (ALP), and gamma-glutamyl transpeptidase (GGT). The platelet count to spleen diameter ratio (PC/SD) was calculated from the platelet count and spleen diameter. The Child-Pugh score was calculated from the hepatic encephalopathy and ascites statuses, total bilirubin, PT, and albumin levels (Cholongitas et al., 2005). The Model for End-Stage Liver Disease (MELD) score was calculated from creatinine, total bilirubin, and INR values (Kamath et al., 2007). The FIB-4 score was calculated from the age, AST, ALT, and platelet count (Sterling et al., 2006). The APRI was calculated from AST and platelet count (Wai et al., 2003). The AAR was calculated from the AST and ALT levels (Williams and Hoofnagle, 1988).

### Statistical Analysis

Statistical analysis was performed using SPSS version 25.0 and RStudio version 4.1.2. Kolmogorov–Smirnov tests of normality were performed for continuous variables. Normally distributed continuous variables were presented as mean ± standard deviation (SD) and examined statistically using Student’s *t*-test. Skewed distributed continuous variables were presented as median (lower and upper quartiles) and examined statistically using Mann-Whitney test. Categorical variables were examined using *χ* ^2^-test. Variables showing *p* < 0.05 on univariate analysis were included in a multivariable forward likelihood ratio (LR) stepwise logistic regression. Variables showing *p* < 0.05 were finally selected as independent predictors. We derived models for the prediction of EVs and red signs based on these predictors. Multicollinearity of variables in the models was tested by examining the variance inflation factor. In the models, the variance inflation factor was always less than 2, confirming the absence of significant multicollinearity (Piastra et al., 2011). To assess the diagnostic performance of the models, receiver operating characteristic (ROC) curves were constructed and each area under the ROC curve (AUROC) was computed. Bootstrap estimation of the AUROC (resampling = 1000) was performed as an internal validation using the R package pROC. The corresponding point of the maximum Youden index was selected as the optimal cutoff value, and a positive prediction result was defined as being equal to or greater than the optimal cutoff value. Cross tabulation of the prediction models was performed based on the results of endoscopic examination. The sensitivity, specificity, positive predictive value (PPV), negative predictive value (NPV), and accuracy were calculated using cross tabulation. There was no missing data on examination results or prediction model results. All statistical analysis were two tailed. The value of *α* was set at 0.05.

## Results

### Patient Characteristics

Among 661 patients with HBV-related liver cirrhosis, a total of 356 patients with compensated or decompensated cirrhosis met the inclusion and exclusion criteria, and were finally enrolled in the study (Figure 1). Of these, 288 (80.9%) patients were men with a mean age of 52 ± 11 years. EVs and red signs were present in 310 (87.1%) and 165 (46.3%) patients, respectively. A total of 133 (37.3%) patients were classified as Child-Pugh class A, 148 (41.6%) were class B, and 75 (21.1%) were class C. Ascites was present in 209 (58.7%) patients, and previous variceal bleeding was present in 34 (9.6%) patients. A total of 159 (44.7%) patients had a history of antiviral therapy. The demographic and clinical characteristics of the included patients are shown in Table 1.

**TABLE 1:** Demographic and clinical characteristics of 356 patients.

Variable	Total (*n* = 356)
**EVs (yes/no)**	310 (87.1%)/46 (12.9%)
**Red signs (yes/no)**	165 (46.3%)/191 (53.7%)
**Age (years)**	52 ± 11
**Sex (male/female)**	288 (80.9%)/68 (19.1%)
**Child-Pugh classification (A/B/C)**	133 (37.3%)/148 (41.6%)/75 (21.1%)
**Child-Pugh score**	7.0 (6.0, 9.0)
**MELD score**	11.9 (9.3, 16.4)
**Ascites (yes/no)**	209 (58.7%)/147 (41.3%)
**Previous variceal bleeding (yes/no)**	34 (9.6%)/322 (90.4%)
**Spleen diameter (cm)**	12.83 ± 2.61
**PC/SD**	5.59 (3.73, 8.73)
**Splenic vein diameter (mm)**	9.0 (7.0, 11.0)
**Portal vein diameter (mm)**	12.0 (11.0, 13.0)
**WBC (10^9^/L)**	3.99 (2.92, 5.39)
**RBC (10^12^/L)**	3.66 (3.09, 4.27)
**Platelet count (10^9^/L)**	72.50 (50.00, 96.00)
**Lymphocyte count (10^9^/L)**	1.08 (0.70, 1.41)
**Lymphocyte ratio (%)**	25.95 (20.20, 32.80)
**Hemoglobin (g/L)**	112.5 (94.0, 128.0)
**PT (s)**	16.80 (15.30, 18.80)
**Total bilirubin (μmol/L)**	26.60 (14.73, 56.18)
**Albumin (g/L)**	34.65 ± 5.99
**Globulin (g/L)**	30.1 (26.5, 34.5)
**A/G**	0.88 (0.72, 1.10)
**AST (U/L)**	45.50 (32.00, 76.75)
**ALT (U/L)**	34.00 (23.00, 57.00)
**ALP (U/L)**	101.00 (78.25, 133.75)
**GGT (U/L)**	51.00 (27.00, 93.00)
**FIB-4**	5.81 (3.69, 9.65)
**APRI**	1.77 (1.03, 3.00)
**AAR**	1.33 (1.00, 1.75)

Normally distributed continuous variables are shown as mean ± standard deviation. Skewed distributed continuous variables are shown as median (lower and upper quartiles). Categorical variables are shown as n (%).

AAR, aspartate aminotransferase to alanine aminotransferase ratio; A/G, albumin to globulin ratio; ALP, alkaline phosphatase; ALT, alanine aminotransferase; APRI, aspartate aminotransferase to platelet ratio index; AST, aspartate aminotransferase; EV, esophageal varices; FIB-4, Fibrosis-4 score; GGT, gamma-glutamyl transpeptidase; PC/SD, platelet count to spleen diameter ratio; PT, prothrombin time; RBC, red blood cell count; WBC, white blood cell count.

### Univariate and Multivariate Analysis of Variables According to the Presence of Esophageal Varices

Table 2 shows age, sex, the imaging, ultrasound, and laboratory parameters of patients according to the presence of EVs. In univariate analysis, sex, spleen diameter, PC/SD, splenic vein diameter, portal vein diameter, WBC, platelet count, lymphocyte count, and lymphocyte ratio were significantly different between patients with and without EVs, whereas no differences in age, Child-Pugh score, MELD score, RBC, hemoglobin, PT, total bilirubin, albumin, globulin, A/G, AST, ALT, ALP, GGT, FIB-4, APRI, and AAR were observed. Variables that showed statistical differences in univariate analysis were included in a multivariable forward LR stepwise logistic regression. Spleen diameter, splenic vein diameter, and lymphocyte ratio were statistically significant in multivariate analysis (Table 2).

**TABLE 2:** Univariate and multivariate analysis of variables according to the presence of EVs.

Variable	EV group (*n* = 310)	Non-EV group (*n* = 46)	Univariate analysis (*p* value)	Multivariate analysis (*p* value)
**Age (years)**	51 ± 11	54 ± 11	0.107	—
**Sex (male/female)**	257/53	31/15	**0.013**	0.211
**Child-Pugh score**	7.0 (6.0, 9.0)	8.0 (6.0, 9.3)	0.389	—
**MELD score**	11.8 (9.3, 16.3)	13.7 ± 5.3	0.506	—
**Spleen diameter (cm)**	13.16 ± 2.53	10.58 ± 1.99	**<0.001**	**<0.001**
**PC/SD**	5.36 (3.57, 7.89)	9.32 (6.04, 12.51)	**<0.001**	0.539
**Splenic vein diameter (mm)**	9.0 (7.0, 11.0)	6.0 (6.0, 8.0)	**<0.001**	**0.003**
**Portal vein diameter (mm)**	12.0 (11.0, 13.8)	12.0 (10.2, 12.0)	**<0.001**	0.079
**WBC (10** ^**9**^ **/L)**	3.88 (2.85, 5.22)	4.73 (3.51, 6.39)	**0.004**	0.429
**RBC (10** ^**12**^ **/L)**	3.64 (3.04, 4.27)	3.80 ± 0.91	0.507	—
**Platelet count (10** ^**9**^ **/L)**	70.00 (48.75, 93.00)	94.00 (63.75, 122.50)	**<0.001**	0.797
**Lymphocyte count (10** ^**9**^ **/L)**	1.00 (0.68, 1.34)	1.43 ± 0.49	**<0.001**	0.531
**Lymphocyte ratio (%)**	25.40 (19.90, 31.60)	31.20 (24.20, 36.45)	**0.002**	**<0.001**
**Hemoglobin (g/L)**	112.0 (93.0, 128.0)	116.0 (101.0, 126.8)	0.299	—
**PT (s)**	16.80 (15.30, 18.80)	17.50 ± 3.26	0.880	—
**Total bilirubin (μmol/L)**	25.65 (14.60, 53.80)	33.45 (18.53, 73.43)	0.109	—
**Albumin (g/L)**	34.85 (31.00, 38.30)	33.80 (30.08, 38.08)	0.585	—
**Globulin (g/L)**	30.50 (26.68, 34.63)	28.50 (25.55, 33.55)	0.127	—
**A/G**	0.89 (0.72, 1.10)	0.81 (0.71, 1.18)	0.594	—
**AST (U/L)**	45.00 (32.00, 74.00)	55.50 (32.50, 91.50)	0.167	—
**ALT (U/L)**	33.50 (23.00, 54.00)	36.00 (22.00, 106.25)	0.480	—
**ALP (U/L)**	100.00 (79.00, 132.00)	109.42 ± 42.15	0.995	—
**GGT (U/L)**	51.00 (27.00, 90.50)	59.50 (30.75, 116.50)	0.279	—
**FIB-4**	6.04 (3.87, 9.60)	5.10 (3.16, 10.02)	0.304	—
**APRI**	1.79 (1.07, 2.95)	1.52 (0.83, 3.58)	0.537	—
**AAR**	1.32 (1.00, 1.72)	1.44 (0.95, 1.90)	0.646	—

Normally distributed continuous variables are shown as mean ± standard deviation. Skewed distributed continuous variables are shown as median (lower and upper quartiles). Categorical variable, sex, is shown as absolute numbers. Bold indicates p value < 0.05.

AAR, aspartate aminotransferase to alanine aminotransferase ratio; A/G, albumin to globulin ratio; ALP, alkaline phosphatase; ALT, alanine aminotransferase; APRI, aspartate aminotransferase to platelet ratio index; AST, aspartate aminotransferase; EV, esophageal varices; FIB-4, Fibrosis-4 score; GGT, gamma-glutamyl transpeptidase; PC/SD, platelet count to spleen diameter ratio; PT, prothrombin time; RBC, red blood cell count; WBC, white blood cell count.

### Prediction Model for Esophageal Varices and Evaluation of Diagnostic Performance

Based on multivariate analysis results above, spleen diameter, splenic vein diameter, and lymphocyte ratio were maintained as independent predictors in the final model (Table 3). We then derived the following spleen diameter-splenic vein diameter-lymphocyte ratio-EV (SSL-EV) prediction model based on the regression coefficients:

SSL-EV=0.416×(spleen diameter)(cm)+0.289×(splenic vein diameter)(mm)−0.054×(lymphocyte ratio)(%)−3.690.

**TABLE 3:** Variables used to establish the prediction model for the presence of EVs.

Variable	B	SE	Wald	*p* Value	OR	95% CI of OR
**Spleen diameter**	0.416	0.093	20.039	<0.001	1.516	1.264∼1.820
**Splenic vein diameter**	0.289	0.098	8.690	0.003	1.355	1.102∼1.617
**Lymphocyte ratio**	−0.054	0.015	12.262	<0.001	0.948	0.920∼0.977
**Constant**	−3.690	1.112	11.009	0.001	0.025	—

B, regression coefficient; CI, confidence interval; OR, odds ratio; SE, standard error.

**FIGURE 2:**
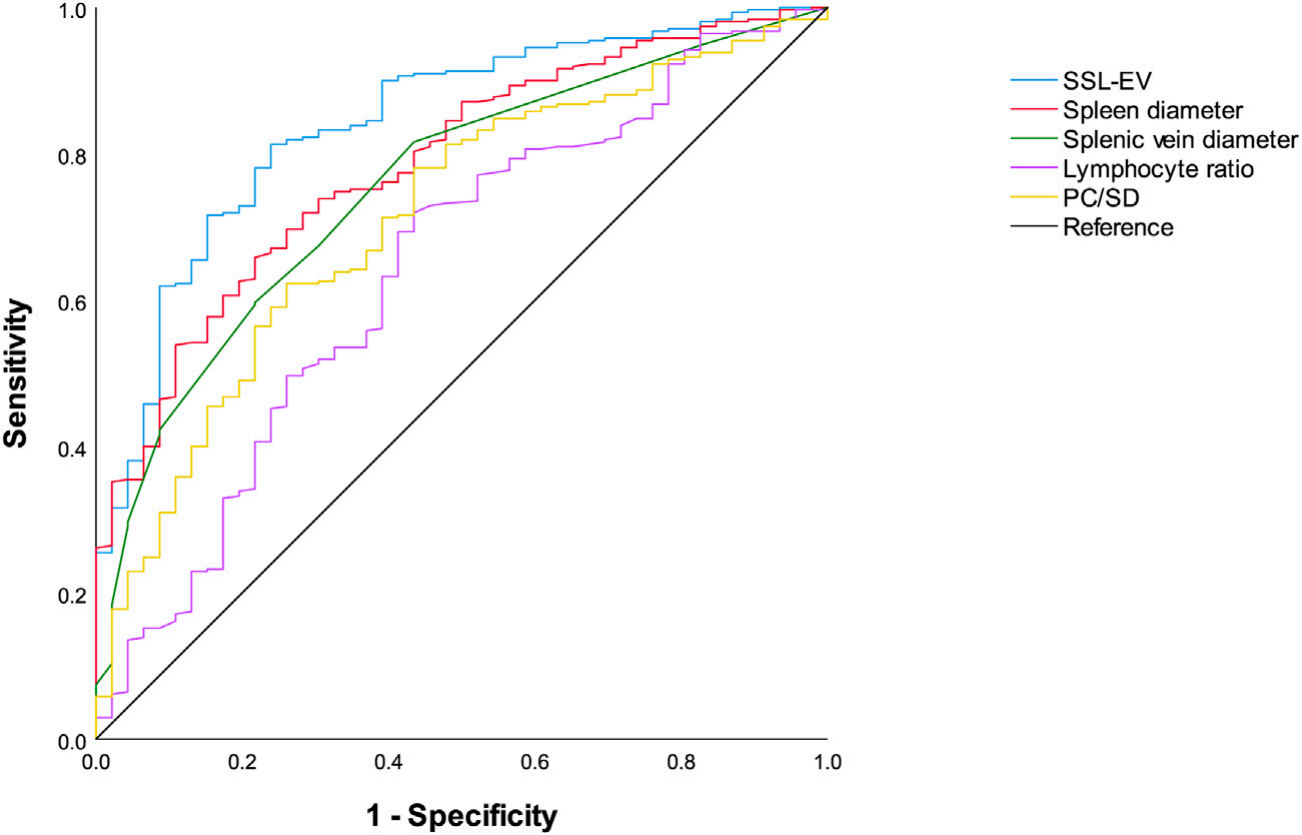
Receiver operating characteristics curves of SSL-EV prediction model, spleen diameter, splenic vein diameter, lymphocyte ratio, and PC/SD for the diagnosis of EVs. SSL-EV prediction model, spleen diameter-splenic vein diameter-lymphocyte ratio-esophageal varices prediction model; PC/SD, platelet count to spleen diameter ratio.

The ROC curves for the prediction of the presence of EVs by SSL-EV and each single predictor are shown in Figure 2. Since previous studies have indicated that PC/SD is an important predictor of EVs, we included PC/SD in the ROC curves. The AUROC (95% CI) of SSL-EV was 0.843 (0.785–0.901), showing the superiority of the diagnostic accuracy over single predictors: spleen diameter, 0.786 (0.723–0.849); splenic vein diameter, 0.756 (0.686–0.825); lymphocyte ratio, 0.642 (0.554–0.731); and PC/SD, 0.713 (0.636–0.790). The AUROC (95% CI) obtained by internal validation using the bootstrap method were as follows: SSL-EV, 0.842 (0.841–0.843); spleen diameter, 0.786 (0.785–0.787); splenic vein diameter, 0.756 (0.755–0.757); lymphocyte ratio, 0.642 (0.641–0.643); PC/SD, 0.713 (0.711–0.714).

The optimal cutoff value of SSL-EV was 1.55 with 81.3% (252/310) sensitivity, 76.1% (35/46) specificity, 95.8% (252/263) PPV, 37.6% (35/93) NPV, and 80.6% (287/356) accuracy. Using this cutoff value, 81.3% patients with EVs could be accurately diagnosed, and the diagnosis could be accurately excluded in 76.1% patients without EVs.

### Univariate and Multivariate Analysis of Variables According to the Presence of Red Signs

As shown in Table 4, patients with red signs had a significantly higher spleen diameter, splenic vein diameter, and portal vein diameter, and lower age, PC/SD, WBC, platelet count, lymphocyte count, lymphocyte ratio, hemoglobin, AST, ALP, and GGT levels than patients without red signs. No differences were observed in sex, Child-Pugh score, MELD score, RBC, PT, total bilirubin, albumin, globulin, A/G, ALT, FIB-4, APRI, and AAR. Variables showing statistical differences in univariate analysis were included in a multivariable forward LR stepwise logistic regression. Spleen diameter, splenic vein diameter, and lymphocyte ratio had statistical significance in multivariate analysis (Table 4).

**TABLE 4:** Univariate and multivariate analysis of variables according to the presence of red signs.

Variable	Red signs group (*n* = 165)	Non-red signs group (*n* = 191)	Univariate analysis (*p* value)	Multivariate analysis (*p* value)
**Age (years)**	50 ± 11	53 (44, 60)	**0.046**	0.905
**Sex (male/female)**	139/26	149/42	0.136	—
**Child-Pugh score**	7.0 (6.0, 9.0)	7.0 (6.0, 9.0)	0.957	—
**MELD score**	11.6 (9.2, 15.9)	12.2 (9.5, 17.2)	0.167	—
**Spleen diameter (cm)**	14.10 (12.10, 15.28)	11.87 ± 2.18	**<0.001**	**<0.001**
**PC/SD**	4.96 (3.19, 7.00)	6.62 (4.33, 9.83)	**<0.001**	0.573
**Splenic vein diameter (mm)**	10.0 (8.0, 11.5)	7.0 (6.0, 10.0)	**<0.001**	**0.004**
**Portal vein diameter (mm)**	13.0 (11.5, 14.0)	12.0 (11.0, 13.0)	**<0.001**	0.478
**WBC (10** ^**9**^ **/L)**	3.69 (2.55, 5.35)	4.21 (3.04, 5.52)	**0.020**	0.712
**RBC (10** ^**12**^ **/L)**	3.66 ± 0.88	3.71 (3.17, 4.28)	0.179	—
**Platelet count (10** ^**9**^ **/L)**	67.00 (45.00, 87.00)	77.00 (53.00, 103.00)	**0.002**	0.878
**Lymphocyte count (10** ^**9**^ **/L)**	0.84 (0.61, 1.21)	1.21 (0.89, 1.57)	**<0.001**	0.841
**Lymphocyte ratio (%)**	23.33 (18.65, 29.85)	28.80 (22.30, 36.00)	**<0.001**	**<0.001**
**Hemoglobin (g/L)**	104.9 ± 25.9	116.0 (101.0, 130.0)	**<0.001**	0.058
**PT (s)**	16.80 (15.10, 18.80)	16.80 (15.30, 19.00)	0.563	—
**Total bilirubin (μmol/L)**	24.36 (14.35, 44.75)	28.20 (16.80, 69.00)	0.052	—
**Albumin (g/L)**	34.92 ± 5.09	34.42 ± 6.68	0.428	—
**Globulin (g/L)**	30.10 (26.05, 33.75)	30.10 (26.50, 35.60)	0.103	—
**A/G**	0.87 (0.72, 1.02)	0.89 (0.72, 1.21)	0.119	—
**AST (U/L)**	42.00 (30.50, 63.00)	53.00 (33.00, 87.00)	**0.004**	0.596
**ALT (U/L)**	31.00 (23.00, 48.00)	37.00 (24.00, 77.00)	0.052	—
**ALP (U/L)**	95.00 (74.00, 122.50)	107.00 (81.00, 145.00)	**0.012**	0.208
**GGT (U/L)**	42.00 (23.00, 79.00)	57.00 (31.00, 107.00)	**0.001**	0.207
**FIB-4**	5.76 (3.59, 9.88)	5.86 (3.77, 9.64)	0.966	—
**APRI**	1.64 (0.99, 2.89)	1.89 (1.05, 3.14)	0.336	—
**AAR**	1.34 (0.98, 1.70)	1.33 (1.00, 1.81)	0.932	—

Normally distributed continuous variables are shown as mean ± standard deviation. Skewed distributed continuous variables are shown as median (lower and upper quartiles). Categorical variable, sex, is shown as absolute numbers. Bold indicates p value < 0.05.

AAR, aspartate aminotransferase to alanine aminotransferase ratio; A/G, albumin to globulin ratio; ALP, alkaline phosphatase; ALT, alanine aminotransferase; APRI, aspartate aminotransferase to platelet ratio index; AST, aspartate aminotransferase; FIB-4, Fibrosis-4 score; GGT, gamma-glutamyl transpeptidase; PC/SD, platelet count to spleen diameter ratio; PT, prothrombin time; RBC, red blood cell count; WBC, white blood cell count.

### Prediction Model for Red Signs and Evaluation of Diagnostic Performance

Spleen diameter, splenic vein diameter, and lymphocyte ratio were selected as independent predictors of red signs on the basis of multivariate analysis results above (Table 5). A new prediction model for red signs called the spleen diameter-splenic vein diameter-lymphocyte ratio-red signs (SSL-RS) prediction model, was proposed according to the following regression coefficients:

SSL-RS=0.320×(spleen diameter)(cm)+0.158×(splenic vein diameter)(mm)−0.067×(lymphocyte ratio)(%)−3.866.

**TABLE 5:** Variables used to establish the prediction model for the presence of red signs.

Variable	B	SE	Wald	*p* Value	OR	95% CI of OR
**Spleen diameter**	0.320	0.061	27.840	<0.001	1.377	1.223∼1.551
**Splenic vein diameter**	0.158	0.055	8.162	0.004	1.171	1.051∼1.304
**Lymphocyte ratio**	−0.067	0.014	24.061	<0.001	0.935	0.911∼0.961
**Constant**	−3.866	0.778	24.683	<0.001	0.021	—

B, regression coefficient; CI, confidence interval; OR, odds ratio; SE, standard error.

**FIGURE 3:**
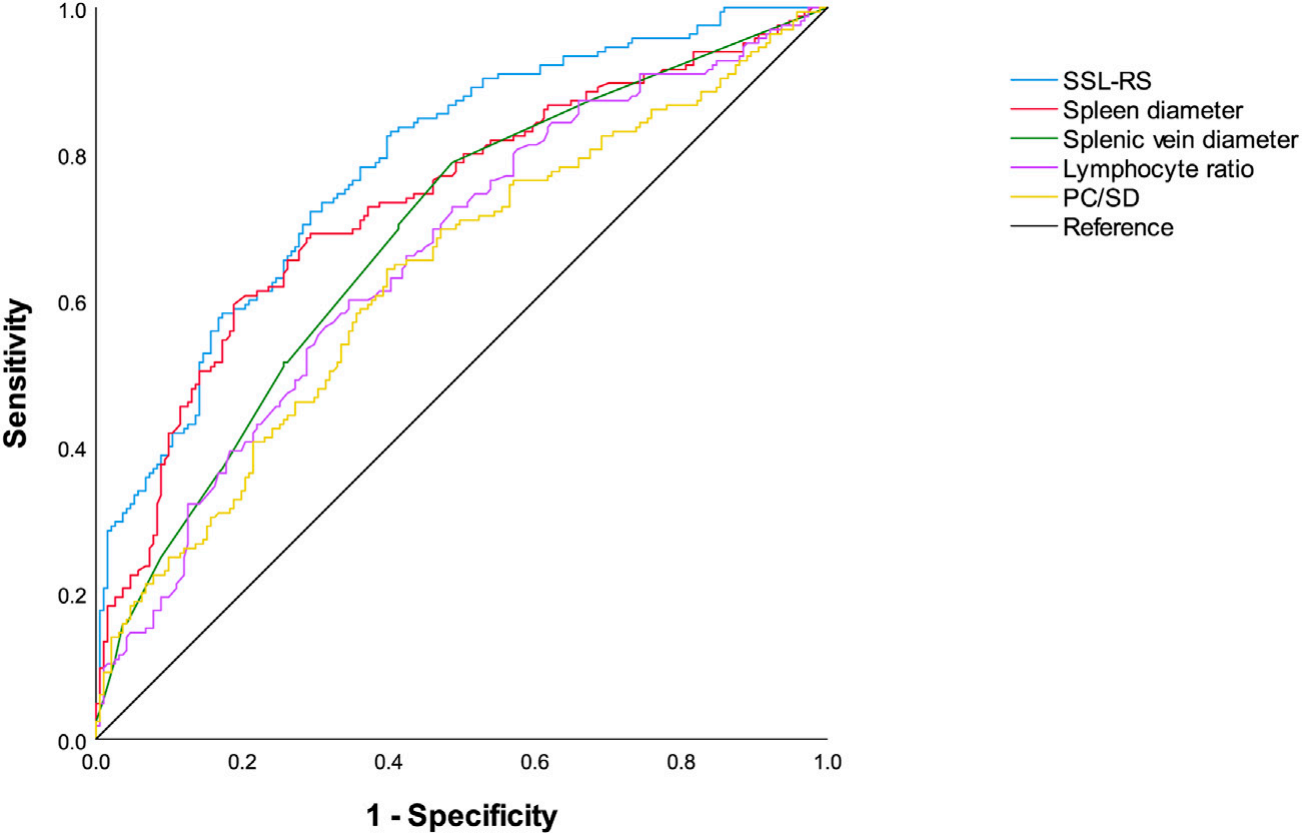
Receiver operating characteristics curves of SSL-RS prediction model, spleen diameter, splenic vein diameter, lymphocyte ratio, and PC/SD for the diagnosis of red signs. SSL-RS prediction model, spleen diameter-splenic vein diameter-lymphocyte ratio-red signs prediction model; PC/SD, platelet count to spleen diameter ratio.

The ROC curves for the prediction of the presence of red signs by SSL-RS, each single predictor, and PC/SD are shown in Figure 3. The AUROC (95% CI) were as follows: SSL-RS, 0.783 (0.736–0.830); spleen diameter, 0.733 (0.680–0.785); splenic vein diameter, 0.686 (0.631–0.741); lymphocyte ratio, 0.659 (0.603–0.716); PC/SD, 0.633 (0.575–0.691). The AUROC (95% CI) obtained by internal validation using the bootstrap method were 0.783 (0.782–0.784) for SSL-RS, 0.733 (0.732–0.734) for spleen diameter, 0.686 (0.685–0.687) for splenic vein diameter, 0.659 (0.658–0.660) for lymphocyte ratio, and 0.633 (0.632–0.634) for PC/SD. The SSL-RS achieved the highest diagnostic accuracy, according to the AUROC.

The optimal cutoff value for the SSL-RS was -0.20. The sensitivity, specificity, PPV, NPV, and accuracy of the optimal cutoff value of the SSL-RS were 72.1% (119/165), 70.7% (135/191), 68.0% (119/175), 74.6% (135/181), and 71.3% (254/356), respectively. The presence of red signs was accurately diagnosed in 72.1% of the patients, and the absence of red signs was confirmed in 70.7% of the patients.

## Discussion

As the second most common decompensation event with high mortality in patients with cirrhosis, EVB imposes a heavy economic burden on society and patients (Garcia-Tsao et al., 2007). Red signs are closely related to EVB, as its presence in medium/large EVs further increases the risk of EVB. Even small EVs were considered HREVs if red signs were present. Consequently, it is suggested that patients are at a higher risk of EVB once red signs occur, and active treatments should be considered (de Franchis and Baveno, 2015; European Association for the Study of the Liver, 2018). Moreover, the presence of red signs in small EVs increases the risk of progression to large EVs (World Gastroenterology Organisation, 2014). It is necessary to predict the presence of red signs in patients with cirrhosis to prevent the occurrence of EVB and progression of small EVs. Owing to the lack of widespread clinical application of endoscopy and FibroScan in primary medical institutions (Tripathi et al., 2015), prediction models for the presence of red signs and EVs non-invasively, conveniently, and reproducibly are needed. Although CT and MRI can detect the presence of EVs in most cases, the presence of red signs or EVB cannot be detected. To the best of our knowledge, non-invasive prediction of the presence of red signs has not been reported in the literature. This research gap is expected to be filled by our study.

**FIGURE 4:**
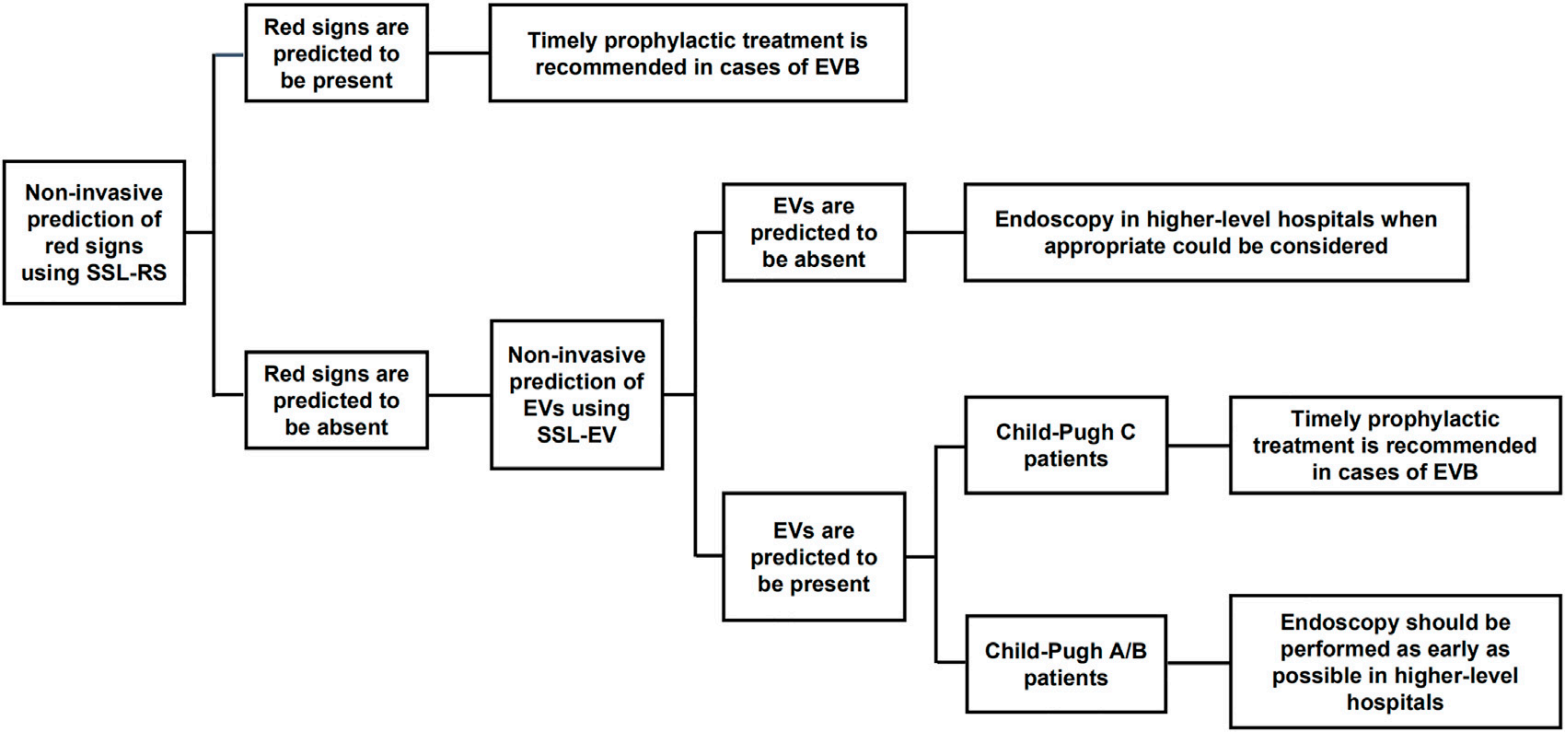
The combination of SSL-RS and SSL-EV prediction models contributes to better decision making in clinical practice. SSL-RS prediction model, spleen diameter-splenic vein diameter-lymphocyte ratio-red signs prediction model; SSL-EV prediction model, spleen diameter-splenic vein diameter-lymphocyte ratio-esophageal varices prediction model.

In this study, the analysis of imaging, ultrasonographic, and laboratory parameters of 356 patients with HBV-related liver cirrhosis indicated that spleen diameter, splenic vein diameter, and lymphocyte ratio were independent risk factors for EVs and red signs. The prediction model of red signs (SSL-RS) based on the parameters above, presented an AUROC of 0.783 (0.736–0.830), 72.1% sensitivity, and 70.7% specificity, while the prediction model of EV (SSL-EV) presented an AUROC of 0.843 (0.785–0.901), 81.3% sensitivity, and 76.1% specificity. The combination of these two prediction models before performing endoscopy would contribute to better decision making in clinical practice, especially in primary medical institutions (Figure 4). If red signs are predicted, timely prophylactic treatment is recommended in cases of EVB. If neither red signs nor EVs are predicted to be present, endoscopy in higher-level hospitals when appropriate could be considered. If red signs are predicted to be absent and EVs are predicted to be present, Child-Pugh classification of the patient requires consideration, since Child-Pugh class C cirrhosis also increases the risk of EVB (de Franchis and Baveno, 2015; European Association for the Study of the Liver, 2018). Prophylactic intervention should be undertaken as early as possible in Child C patients, and endoscopy should be performed as early as possible in Child A/B patients, since the presence of medium/large EVs is still possible.

The prediction models used in this study can be calculated easily. The inter-observer variability of the spleen diameter and splenic vein diameter is low, which makes the prediction models reproducible (Sebastianes et al., 2010; Gunes and Akturk, 2018). The measurement of lymphocyte ratio by routine blood tests is easy, highly reproducible, and low-cost (Feng et al., 2018; Chisthi, 2020). Therefore, the prediction models are clinically useful. Additionally, the participants of this study were restricted to patients with HBV-related liver cirrhosis, which makes this study more targeted.

Spleen diameter and splenic vein diameter are associated with cirrhotic portal hypertension. Hypertension of the splenic vein leads to an increased diameter. This causes congestion in the spleen, and consequently, splenomegaly (Merli et al., 2003). Studies have shown that the splenic vein diameter of patients with cirrhosis with HREVs is significantly higher than that of patients with low-risk EVs or healthy controls (Jamil et al., 2017). Previous studies have indicated that PC/SD is an important predictor of EVs, and that the diagnostic performance of PC/SD is better than that of spleen diameter (Giannini et al., 2003; Giannini et al., 2006; Berzigotti et al., 2013; Xu et al., 2016). However, in this study, both the SSL-EV prediction model and spleen diameter yielded a larger AUROC than PC/SD in predicting the presence of EVs. This result is consistent with that of a previous study by Hong et al. (2009), in which spleen width achieved better predictive accuracy than platelets/spleen width in predicting the presence of EVs in patients with HBV-related cirrhosis (AUROC: 0.736 ± 0.049 vs. 0.7095 ± 0.0488). The reasons for the differences in the results could be manifold. The study population or race could affect the spleen diameter. Different etiologies, such as alcoholic cirrhosis (Kothari et al., 2019), HCV-related cirrhosis (Morishita et al., 2014), and schistosomiasis-related liver cirrhosis (Bolondi et al., 1991), may be another reason. Studies have suggested that splenomegaly caused by hepatitic cirrhosis is more severe than splenomegaly caused by alcoholic cirrhosis (Kashani et al., 2015). Furthermore, other factors, including toxic effects on the bone marrow induced by drugs or cirrhosis, and reduced thrombopoietin synthesis, could also lead to a decreased platelet count (Kountouras, 2004; Kumar et al., 2020).

Although the relationship between the lymphocyte ratio and EVs has not been reported, previous studies have demonstrated that the lymphocyte count or ratio is associated with HBV-related cirrhosis. Compared with healthy controls, both the lymphocyte count and lymphocyte/monocyte ratio decreased significantly (Zhang et al., 2015; Zhao et al., 2017; Zhang et al., 2020), and the neutrophil/lymphocyte ratio was markedly elevated (Zhao et al., 2017; Zhang et al., 2020). This may be a consequence of chronic systemic inflammation, immune dysfunction, and T-cell exhaustion due to chronic HBV infection (Lombardo et al., 1995; Ye et al., 2015; Kalra et al., 2017). Additionally, the poor nutritional status of patients with decompensated cirrhosis may contribute to the decreased lymphocyte count/ratio (O'Keefe et al., 1980; Rocha and Fortes, 2015). This study revealed that the lymphocyte ratio was an independent risk factor for EVs and red signs. In addition, the lymphocyte count was significantly lower in patients with EVs and red signs. The occurrence of EVs or red signs indicates that the patients are in their last stage of chronic liver disease, namely, cirrhosis. Severe systemic inflammation, immune dysfunction, and nutritional status of patients in this disease stage are likely to cause a decreased lymphocyte ratio.

This study inevitably suffers from several limitations. This was a retrospective study, with a relatively small sample size. Hence, a prospective study with a larger sample size should be conducted for more in-depth research. The lack of external validation is another limitation since external validation will evaluate the usefulness of the proposed models and whether they have widespread clinical use. In terms of the etiology of cirrhosis, only patients with HBV-related cirrhosis were enrolled in this study. But non-HBV etiologies of cirrhosis also account for a substantial part. Therefore, assessing whether the proposed models could work in non-HBV etiologies of cirrhosis is meaningful and requires further study.

In conclusion, spleen diameter, splenic vein diameter, and lymphocyte ratio were independent predictors of EVs and red signs in patients with HBV-related liver cirrhosis. Based on these predictors, a non-invasive prediction model for red signs was first proposed. Together with the prediction model for EVs proposed here, more targeted decision making in clinical practice in primary medical institutions could be expected. It would be beneficial to avoid the occurrence of EVB, and consequently improve the prognosis of patients with HBV-related cirrhosis.

## Data Availability

The original contributions presented in the study are included in the article/supplementary material, further inquiries can be directed to the corresponding authors.
